# The Relationship between Emotional Intelligence and Cool and Hot Cognitive Processes: A Systematic Review

**DOI:** 10.3389/fnbeh.2016.00101

**Published:** 2016-05-27

**Authors:** María José Gutiérrez-Cobo, Rosario Cabello, Pablo Fernández-Berrocal

**Affiliations:** ^1^Department of Basic Psychology, Faculty of Psychology, University of MálagaMalaga, Spain; ^2^Department of Developmental and Educational Psychology, University of GranadaGranada, Spain

**Keywords:** emotional intelligence, cognitive processes, hot tasks, cool tasks

## Abstract

Although emotion and cognition were considered to be separate aspects of the psyche in the past, researchers today have demonstrated the existence of an interplay between the two processes. Emotional intelligence (EI), or the ability to perceive, use, understand, and regulate emotions, is a relatively young concept that attempts to connect both emotion and cognition. While EI has been demonstrated to be positively related to well-being, mental and physical health, and non-aggressive behaviors, little is known about its underlying cognitive processes. The aim of the present study was to systematically review available evidence about the relationship between EI and cognitive processes as measured through “cool” (i.e., not emotionally laden) and “hot” (i.e., emotionally laden) laboratory tasks. We searched Scopus and Medline to find relevant articles in Spanish and English, and divided the studies following two variables: cognitive processes (hot vs. cool) and EI instruments used (performance-based ability test, self-report ability test, and self-report mixed test). We identified 26 eligible studies. The results provide a fair amount of evidence that performance-based ability EI (but not self-report EI tests) is positively related with efficiency in hot cognitive tasks. EI, however, does not appear to be related with cool cognitive tasks: neither through self-reporting nor through performance-based ability instruments. These findings suggest that performance-based ability EI could improve individuals’ emotional information processing abilities.

## Introduction

Despite the crucial role that emotions play in our lives, their mechanics are still not properly understood. What is accepted in the research community is that emotions imply physiological, cognitive, and behavioral changes (Lewis et al., 2008), as well as that they have both positive and negative valences. Thus, if we imagine someone going into her office and seeing a snake on her desk, unless she is a lover of snakes, she will feel frightened (negative valence). She will then express fear at three levels: the physiological (e.g., an increase in heart rate), cognitive (e.g., thoughts about danger), and behavioral (e.g., the urge to run away) levels.

Emotions and cognition have been understood to be different, and even incompatible, aspects of the human psyche in the past. Nonetheless, today the scientific evidence shows that emotions have an important influence on our cognitive processing, and that a balance between cognition and emotion could be the best strategy for correct environmental and social adaptation (Ekman, 1989; Lazarus, 1991; Damasio, 1994; LeDoux, 1996; Keltner and Haidt, 2001; Barrett, 2013). Emotional Intelligence (EI) is a relatively new concept that try to connect the emotion and cognition concepts since 25 years ago (Salovey and Mayer, 1990). Mayer and Salovey (1997, pp. 3–31) have defined this construct as:

… the ability to perceive accurately, appraise, and express emotion; the ability to access and/or generate feelings when they facilitate thought; the ability to understand emotion and emotional knowledge; and the ability to regulate emotions to promote emotional and intellectual growth.

Researchers have traditionally conceptualized EI following two theoretical approaches: mixed and ability models (Mayer et al., 2008). Joseph and Newman (2010) have recently proposed a new division: the authors suggest theoretically classifying the EI construct into three perspectives, paying attention to the kind of instrument that is employed for measuring the construct: performance-based ability EI, self-report ability EI, and self-report mixed EI. *Performance-Based Ability Models* assess EI through performance tests, and they conceive EI as a narrow cognitive concept, as well as a kind of intelligence that is based on a set of emotional aptitudes (Mayer et al., 2000). In such measures, participants must solve emotional problems in which there are better and worse responses. The Mayer-Salovey-Caruso Emotional Intelligence Test (MSCEIT; Mayer et al., 2002) is the most important performance test of EI; it is based on a hierarchical ability EI model (Mayer and Salovey, 1997). *Self-Report Mixed Models* aim for a broader construct; they are measured through self-report instruments, which include mental abilities, personality factors, motivations, interpersonal and intrapersonal abilities, and other facets. One of the most representative self-report mixed scales is the Bar-On Emotional Quotient Inventory (EQ-i; Bar-On, 2004). Finally, *Self-Report Ability Models*, although they also assess EI through self-reporting, are based on the ability EI model. The Trait Meta-Mood Scale (TMMS; Salovey et al., 1995) is a well-known instrument for this group. Both self-report mixed and self-report ability scales evaluate the subjective perception that participants have about their own EI. In such scales, there are no correct or incorrect responses.

Researchers have related EI measured through mixed and ability self-reporting and performance-based ability models to a wide range of outcomes. Scholars have found evidence of the relation between EI in mental and physical health (e.g., Schutte et al., 2007; Martins et al., 2010; Zeidner et al., 2012); with less aggressive behavior (García-Sancho et al., 2014), with substance abuse (Kun and Demetrovics, 2010); and with academic (Hansenne and Legrand, 2012; Dolores et al., 2013) and job performance (Joseph and Newman, 2010; Côté, 2014), among other factors. A longitudinal study, using a cognitive Go/No-Go task with hot and cool stimuli as well as brain measures (Casey et al., 2011), has shown how impulsive children appear to have lower emotion regulation and lower self-control abilities in their mid-forties as compared with low impulsive children. As opposed to this broad background, less is known about the cognitive processes underlying EI. EI could favor to manage, in a more proper way, our cognitive resources. For instance, training EI abilities may help to diminish the negative bias of depressed people towards neutral stimuli (Baddeley, 2007) and of non-depressed people after a negative mood induction (Baddeley et al., 2012) by perceiving emotions and situations in a more positive way. Besides, EI training could improve the individual’s cognitive capacity by reducing the interference that anxiety may exert in their performance by improving the emotional regulatory strategies (Derakshan and Eysenck, 1998) as well as by increasing the low threshold that anxious people have for detecting a threat (Mogg and Bradley, 1998, 2005).

In spite of the previous evidence of studies that connect the EI construct with a large number of daily outcomes, researchers have largely criticized these efforts. Some argue that EI is a conglomeration of old concepts that have already been studied; some also argue that it cannot be understood as a form of intelligence (Locke, 2005). In order to address this criticism, several researchers have attempted to prove that EI is a form of intelligence by analyzing its relationship with conventional psychometric intelligence measures (e.g., Roberts et al., 2001; O’Connor and Little, 2003; MacCann et al., 2004). These studies show a positive correlation between performance based ability measures of EI and conventional intelligence, without endangering its singularity (Kong, 2014). One way to take a step forward in the conceptualization of EI in the intelligence domain (and to achieve a better theoretical understanding of the nature of the construct) would be to look for evidence of the relationship of EI to cognitive processes that have been evaluated by using laboratory tasks, instead of using traditional tests. It would also be important to know that cognitive tasks could be divided (depending on the kind of stimuli that are used), as well as the kinds of consequences that participants undergo when performing hot and cool tasks. Thus, we refer to tasks as being “hot” when they contain affective or emotional stimuli, or when the outcome can be a reward or a punishment, and to tasks as being “cool” when the stimuli are emotionally neutral (Denham et al., 2012; Allan and Lonigan, 2014).

## The Present Study

The aim of the present study is to systematically review the existing evidence about the relationship between the EI construct and different cognitive processes, measured by computer laboratory tasks such as (for instance) the Iowa gambling task (IGT; Lin et al., 2013). We expect that, if EI is a form of intelligence, those with higher skills in this domain should perform better in the different cognitive tasks compared with low-EI individuals when EI is measured through an objective measure, as is the case with performance-based ability models. We also expect to find this advantage for higher-EI individuals in the case of hot tasks, given the emotional nature of the EI construct.

## Materials and Methods

### Literature Search and Inclusion Criteria

The MEDLINE and Scopus databases were carefully searched for suitable articles to use. We selected relevant articles when they contained “EI” as a keyword or as a term in the abstract, together with one or more of the following terms: “behavioral measure,” “abstract thinking,” “attention,” “cognitive ability,” “cognitive flexibility,” “cognitive processes,” “decision making,” “executive function,” “go-nogo task,” “IGT,” “priming,” “reaction time (RT),” “reasoning,” “Stroop,” and “working memory.”

In order to be included in the present systematic review, articles first had to measure EI through a performance-based ability test, a self-report mixed model, or a self-report ability model. Second, cognitive processes had to be assessed through laboratory tasks, and not by traditional testing. Third, participants could not suffer from mental problems; finally, the language of the studies had to be in English or Spanish. Articles were excluded if any of these criteria were not met.

### EI Instruments

#### Performance-based Ability Models

The aforementioned MSCEIT (Mayer et al., 2002) is a 141-item test that assesses the four branches of EI (confirmed through factor analysis; Mayer et al., 2003): identifying, facilitating, understanding, and managing emotions. It presents a test-retest reliability of *r*_(60)_ = 0.86 (Brackett and Mayer, 2001) and a full-test split-half reliability of *r*_(1985)_ = 0.93 and 0.91 for general and expert scoring, respectively (Mayer et al., 2003). The Test of Emotional Intelligence (TEMINT; Schmidt-Atzert and Bühner, 2002) is a 12-situation test, with a Cronbach’s *α* of 0.77, where understanding of emotions is evaluated. Not to be confused with the TEMINT, the Emotional Intelligence Test (TIE, from the Polish *Test Inteligencji Emocjonalnej,B84*) is a 21-item test that measures the four branches of EI grouped as follow due to confirmatory factor analyses (Smieja et al., 2014): first, the perception and understanding of emotions, and second, the facilitation and management of emotions. The overall reliability of this test is *r* = 0.88 with a Cronbach’s *α* of 0.88 for the first part and 0.78 for the second one.

The Situational Judgement Test (SJT; Roberts, 2009) and the SJT of emotional abilities (SJTEA; Roberts et al., 2013) consists of 16 short video clips with one particular scenario that is emotionally laden. This instrument measures emotion management and its internal consistency and its test-retest reliability is 0.61 and 0.54, respectively (MacCann et al., 2015). Ovsyannikova and Lyusin (2009) video test consists of seven short videos that measure emotion recognition using a set of 15 scales of emotion categories per video. This test is made up of an accuracy and a sensitivity index with a Cronbach’s *α* of 0.74 and 0.93, respectively, and a test-retest reliability of 0.55 for accuracy and of 0.86 for sensitivity.

#### Self-report Mixed Models

The EQ-i (Bar-On, 1997) is a 133-item instrument that is comprised of five scales obtained through a factorial validation method: intrapersonal, interpersonal, adaptability, stress management, and general mood. The overall internal consistency coefficient is 0.97 and the test-retest reliability is 0.79. The Shortened Emotional Quotient Inventory (EQ-i:S; Bar-On, 2002) is a shortened version of the EQ-i (with 51 items) that measures total EI with an internal consistency of 0.70, as well as the same five scales of the longer version.

Cooper and Sawaf (1998) EQ_tm_Map includes 259 items that describe five central zones of EI: surround (*α* = 0.87), emotional awareness (*α* = 0.85), dexterity (*α* = 0.89), EQ values and beliefs (*α* = 0.84) and results (*α* = 0.92). The Trait Emotional Intelligence Questionnaire (TEIQue; Petrides and Furnham, 2003) includes 153 items with a Cronbach’s *α* of 0.89 and with a factor analyses that offer four factors: well-being, self-control, emotionality, and sociability. Kemp et al. (2005) Brain Resource Inventory for Emotional Intelligence Factor (BRIEF) is a 14-item questionnaire composed of three factors: intuition and empathy, social skills and relationship management, and self-concept. Its internal consistency is *r* = 0.68–0.81 and its test-retest reliability is *r* = 0.92.

#### Self-report Ability Models

The Schutte Emotional Intelligence Scale (EIS; Schutte et al., 1998) includes 33 items of one-factor solution with three categories: perceiving and expressing emotions, regulating emotions, and utilizing emotions when solving problems. Its Cronbach’s *α* is 0.90 and its test-retest reliability 0.78. Salovey et al.’s (1995); Trait Meta-Mood Scale (TMMS) is a 30-item scale that assesses three subscales based on factor analyses: attention to feelings (*α* = 0.86) clarity of feelings (*α* = 0.87) and mood repair (*α* = 0.82).

The Self-Rated Emotional Intelligence Scale (SREIS; Brackett et al., 2006) includes 19 items whose factor analyses offer a four factor solution: perception, management, use, and understanding emotions. Its Cronbach’s *α* is 0.77. Finally, Lyusin (2006) Emotional Intelligence Inventory (EmIn) uses five basic scales: recognition of others’ emotions, management of others’ emotions, emotional self-awareness, management of one’s own emotions, and control of emotional expression. Its internal consistency is 0.76 and its retest reliability 0.84.

## Results

Our research identified 26 studies that measured EI a total of 44 times, using 13 different scales; we noticed that the majority of the studies analyzed EI by more than one instrument. Seventeen of these 44 times in which EI was assessed were conducted via performance tests, 16 times through self-report ability tests, and 11 through self-report mixed tests. Eighteen different cognitive tasks were used in the 26 studies; 3 of the 18 tasks were classified as “cool, ” and the remaining 15 as “hot.”

In order to present the results that we reviewed, we will separately consider the studies that were conducted using hot and cool cognitive tasks. Apart from that division, we will also discuss the different studies depending on the kind of EI measure that they employed. The reader will thus find the results classified following two criteria: the EI scale (self-report ability test, self-report mixed test, or performance test) and the emotional load of the cognitive task (hot or cool).

### Hot Cognitive Tasks

#### Self-report Ability Tests

We identified 13 studies where EI was measured through self-report ability tests at the same time that EI was related with hot cognitive processes (Table 1). First, Austin (2004) used an emotional inspection time (IT) task for measuring emotional information processing; this is a discrimination task that includes emotional faces. The duration of the presentation of the face stimuli varies across blocks; the IT performance is assessed as the shortest duration that participants require for processing the given stimuli, and thus properly discriminating it. EI was measured through the aforementioned Schutte EIS. The results showed a positive correlation between the “appraisal of emotion” subfactor of the EIS and the emotional IT task (sad and happy faces). Again using an IT task, Austin (2005) also found a significant and positive correlation between the EIS interpersonal scale and an overall emotional performance score (obtained by combining scores on the happy IT, sad IT, and a facial expression recognition task); however, neither Farrelly and Austin (2007), first experiment nor DeBusk and Austin (2011) found any correlation between EI assessed with the same test and the emotional IT task.

**Table 1:** **Studies using self-report ability emotional intelligence (EI), tests and hot cognitive tasks**.

Study	EI scale	Cognitive task	Sample	Principal results
Austin (2004)	EIS	Sad and happy IT tasks	35 department members and 57 undergraduate students (71 females)	Positive correlation between the appraisal of emotion and the emotional IT task
Austin (2005)	EIS	Happy and sad IT tasks	95 adults (71 females)	Positive correlation between the EIS interpersonal scale and the sum of the happy and sad IT and facial recognition task scores
Farrelly and Austin (2007)	EIS	Sad and happy IT tasks	99 university students (70 females)	No relationship between EI and IT tasks
DeBusk and Austin (2011)	EIS	Happy, angry, and sad IT tasks	87 participants	No relationship between EI and IT tasks
Brabec et al. (2012)	TMMS	IGT	103 undergraduate student (76 females)	No association between EI and the total, nor the net block scores on the behavioral task
Demaree et al. (2010)	EIS	IGT	68 undergraduate students	No association between EI and the total, nor the net block scores on the behavioral task
Webb et al. (2014)	SREIS	IGT	65 participants (32 females)	No relationship between EI and IGT task
Fallon et al. (2014)	TMMS	Simulated arctic rescue scenario	169 participants (110 females)	No association between EI and the decision-making task
Coffey et al. (2003)	TMMS	Emotional and a neutral-word Stroop task	129 undergraduate students (58% female)	Those with high attention to emotions (measured with TMMS, and also with two other scales related to alexithymia) displayed longer reaction times in the task
Fisher et al. (2010)	TMMS	Emotional and a neutral-word Stroop task	88 psychology students (53% female)	A trend for “attention to emotion” was found to be negatively correlated with the neutral and negative conditions of an emotion-word Stroop task
Dodonova and Dodonov (2010)	EmIn	Emotional sensitivity task	277 high school and college students (181 females)	Negative correlation between an RT index related to the correct responses for the “No” responses and three subscales of EI (management of others’ emotions, emotional self-awareness, and management of one’s own emotions) and the two interpersonal and intrapersonal higher-level scales of EI
Dodonova and Dodonov (2012)	EmIn	Emotional sensitivity task	87 undergraduate students	Negative correlation between an RT index related to the correct responses for the “No” responses and three subscales of EI (management of others’ emotions, emotional self-awareness, and management of one’s own emotions) and the two interpersonal and intrapersonal higher-level scales of EI
Fellner et al. (2012)	TMMS	Discrimination learning task	180 psychology students (111 females)	Attention to emotions was found to be a significant predictor of higher error rates in the first blocks; the block learning effect was moderated by the “clarity of emotions” subfactor

*Abbreviations: EIS, Schutte Emotional Intelligence Scale (Schutte et al., 1998); EmIn, Emotional Intelligence Scale (Lyusin, 2006); IGT, Iowa gambling task; IT, inspection time; RT, reaction time; SREIS, Self-Rated Emotional Intelligence Scale (Brackett et al., 2006); TMMS, Trait Meta-Mood Scale (Salovey et al., 1995)*.

Another cognitive task that has been used (and which is related to decision-making processes) is the well-known IGT. Here, participants have to select one hundred different cards from four decks. The four decks contain unequal monetary punishments and rewards; the goal is to obtain as much money as possible. Brabec et al.’s (2012) study, using the IGT and the TMMS for EI, did not find any association between EI and the total scores (nor for the net block scores) on the behavioral task. Demaree et al. (2010) found the same outcomes using the EIS. In the same way, using the SREIS, Webb et al. (2014) did not find any correlation. Fallon et al. (2014) also did not find any relationship between the TMMS and the decision-making task called the “simulated arctic rescue scenario,” where participants had to choose the optimal route in a virtual environment.

With a Stroop task (which measure attentional processes), in which participants have to name the ink color of different stimuli with emotional or neutral valence, Coffey et al. (2003) found that those with higher attention to emotions (measured not only with the TMMS, but also with two other scales related to alexithymia) paid more attention to the emotional content of this cognitive task, as was displayed by their longer RTs. Fisher et al. (2010), however, only found a trend for “attention to emotion” in the TMMS to be negatively correlated with neutral and negative conditions of the same attentional task.

Another cognitive task is the “emotional sensitivity” task. This task measure memory processes and participants have to recognize if a neutral or emotional target face has appeared before during the course of a trial. Using this memory instrument, Dodonova and Dodonov (2012) found a negative correlation between a RT index (the difference between the RT to the emotional faces recognition task vs. a neutral face recognition task) related to the correct responses for the “No” responses (trials where the target had not been presented previously) and three subscales of the EmIn questionnaire (management of others’ emotions, emotional self-awareness, and management of one’s own emotions), as well as the two interpersonal and intrapersonal higher-level scales of the same EI instrument. The same authors had found similar results in a previous study (Dodonova and Dodonov, 2010).

Finally, Fellner et al. (2012) had their participants work on a discrimination learning task with happy, sad, and neutral faces, and found a few correlations with some of the subscales of the TMMS. Specifically, the authors found “attention to emotions” to be a significant predictor of higher error rates in the first blocks, and that the block learning effect was moderated by the “clarity of emotions” subfactor.

#### Self-report Mixed Tests

Focusing on self-report mixed instruments, we identified eight studies that used this kind of measure (Table 2). Austin (2005) and Farrelly and Austin (2007), in their second experiment, examined the relationship between the EQ-i:S and a neutral and a sad IT task, and did not find any correlation between the two. Petrides and Furnham (2003), using a series of videos with neutral faces that culminated in one of the six basic emotions, found that participants who were higher in EI (as assessed by the EQ-i) were faster and required fewer phases for the correct identification of facial emotions. They also found that those with higher EI recognized expressions that registered happiness and surprise faster than low-EI participants.

**Table 2:** **Studies using self-report mixed EI tests and hot cognitive tasks**.

Study	EI scale	Cognitive task	Sample	Principal results
Farrelly and Austin (2007)	EQ-i:S	Sad IT task	199 university students (137 females)	No relationship between EI and IT tasks
Austin (2005)	EQ-i:S	Happy, sad, and IT tasks	95 adults (71 females)	No relationship between EI and IT tasks
Petrides and Furnham (2003)	EQ-i	Changeable emotional faces (videos)	34 psychology students (25 females)	Higher-EI participants were faster and required fewer phases for a correct identification of the face emotions; Those with higher EI recognized expressions of happiness and surprise faster than low-EI participants
Webb et al. (2014)	EQ-i:S	IGT	65 participants (32 females)	No relationship between EI and IGT
Pilárik and Sarmány-Schuller (2009)	EQ_tm_Map	IGT	174 female social work students	Positive association between EI and scores on the “awareness” subscale and on the “EQ value and belief” scale; weak, positive relation on the “dexterity” subscale; negative relation with the “surround” scale
Telle et al. (2011)	TEIQue	Computerized emotional gambling tasks (happy, neutral, or fearful)	103 participants (57% female)	Higher-EI participants on the factors of sociability, social awareness, and the capability for fostering interpersonal relationships performed significantly better than lower-EI participants
Alkozei et al. (2015)	EQ-i	Airport task	62 participants (50% female)	No relationship between EI and the airport task
Mikolajczak et al. (2009)	TEIQue	Emotional word dot probe task	62 psychology student (47 females)	Slower responses in the attentional task for those with high punctuations in the “self-control” factor

*Abbreviations: EQ-i, Emotional Quotient Inventory (Bar-On, 1997); EQ-i:S, Shortened Emotional Quotient Inventory (Bar-On, 2002); IGT, Iowa gambling task; IT, inspection time; TEIQue, Trait Emotional Intelligence Questionnaire (Petrides and Furnham, 2003)*.

In contrast, using the IGT as a cognitive task, Webb et al. (2014) did not find any correlation with the EQ-i:S; indeed, Pilárik and Sarmány-Schuller (2009), using a sample of female social work students, found a positive association between EI measured with the EQ_tm_Map and the IGT, as well as with the scores in the subscale of “awareness” in the “EQ value and belief” scale, and a weak and positive relation on the “dexterity” subscale; they also found a negative relation with the “surround” scale. Telle et al. (2011) used a computerized gambling task where participants had to make several financial decisions after the appearance of an emotional face (happy, neutral, or fearful). The authors used the TEIQue as the EI questionnaire, and found a relationship between EI and decision making: higher-EI participants (in particular, in the factors of sociability, social awareness, and the capability of fostering interpersonal relationships) performed significantly better than lower-EI participants. In another decision-making task, where participants had to decide (in an airport security situation) which emotional facial expressions could represent a possible terrorist, Alkozei et al. (2015) did not find any correlation between the EQ-i and acuity in the airport tasks.

Finally, Mikolajczak et al. (2009) employed an attentional word dot probe task; in this task, participants were required to respond as quickly as possible to a visual probe that appeared in the location of a neutral or emotional stimulus that had been presented previously. They employed the TEIQue, and found slower responses in the attentional task for those with high punctuations in the “self-control” factor.

#### Performance Tests

Our search identified 14 studies that employed performance tests for evaluating EI (Table 3). While Farrelly and Austin (2007) found no relationship between EI measured through the MSCEIT and the emotional IT task in their first experiment, in their second experiment, they found a positive, significant correlation between the MSCEIT scores (except in the “managing” branch) and the sad IT task. DeBusk and Austin (2011), on the other hand, using the TEMINT, found that TEMINT was not a significant predictor of performance in the emotional IT task. In addition, Wojciechowski et al. (2014) attempted to assess the emotional information processing of inconsistent signals through a facial-verbal decoding task (FDT). In these tasks, participants had to indicate if individuals who demonstrated a specific facial expression on the computer could have truthfully said particular sentences; participants were presented with congruent and incongruent trials. The authors used the TIE for measuring EI. They did not find any correlation between EI and the FDT RT, although they did find that EI as a whole was positively related with the processing of emotional expressions in all of the subscales of the FDT (congruent and incongruent trials). They found the same outcomes for the “perception” branch of the EI test, but only with congruent trials. In another study, Jacob et al. (2013) used the German-language version of the MSCEIT (Steinmayr et al., 2011) and a laboratory task using verbal and nonverbal emotional cues (again with congruent and incongruent trials) for measuring perceptual emotional information processing, where participants had to indicate their impressions about the emotional states of speaker; they found a significant negative correlation between the individual mean RT and the “facilitating emotions” branch of the MSCEIT, as well as a significant negative correlation among the RT differences between the emotionally incongruent and congruent conditions and the total EI scores, the “understanding emotions” branch, and the faces task of the “perceiving emotions” branch.

**Table 3:** **Studies using performance EI tests and hot cognitive tasks**.

Study		EI scale	Cognitive task	Sample	Principal results
Farrelly and Austin (2007)	Study 1	MSCEIT	Sad and happy IT tasks	99 university students (70 females)	No relationship between EI and IT tasks
	Study 2	MSCEIT	Sad IT task	199 university students (137 females)	Positive correlation between MSCEIT scores (except for the “managing” branch) and the sad IT task
DeBusk and Austin (2011)		TEMINT	Happy, angry, and sad IT tasks	87 participants	No relationship between EI and IT tasks
Wojciechowski et al. (2014)		TIE	Face-decoding test	210 participants (50% female)	EI positively related with processing of emotional expressions in all subscales of the FDT
Jacob et al. (2013)		MSCEIT	Verbal and nonverbal emotional tasks	40 participants (20 females)	Negative correlation between the RT and the “using emotions” branch; negative correlation among the RT differences between the emotionally incongruent and congruent conditions and the total EI score, the “understanding emotions” branch, and the faces task of the “perceiving emotions” branch
Reis et al. (2007)		MSCEIT	Watson card selection task: social exchange problems	48 under-graduate students	Those with higher EI had faster RT in social exchange problems
Webb et al. (2014)		MSCEIT	IGT	65 participants (32 females)	Positive correlation between the IGT and the MSCEIT total scores and the “facilitating” and “understanding” branches; EI did not significantly predict variances of the decision-making task beyond IQ scores
Fallon et al. (2013)		SJT	Simulated arctic rescue scenario	172 participants (133 females)	No differences in EI and route choice, nor in the easy or difficult trials
Fallon et al. (2014)		SJTEA	Simulated arctic rescue scenario	169 participants (110 females)	No differences in EI and the route choice, nor in the easy or difficult trials; tendency of EI to correlate with accuracy
Alkozei et al. (2015)		MSCEIT	Airport task	62 participants (50% female)	Higher-EI participants performed better than lower-EI participants
Martin and Thomas (2011)		MSCEIT	Emotional and neutral word Stroop tasks	87 under-graduate students	Negative correlation between the RT in the cognitive task and the EI test; EI accounted for incremental variance above a traditional IQ test
Fiori and Antonakis (2012)		MSCEIT	Affective and semantic priming tasks	85 participants (55% female)	No relationship between EI and cognitive tasks
Dodonova and Dodonov (2010)		Video test	Emotional sensitivity task	87 under-graduate students	Negative correlation between an RT index related to the correct responses for the “No” responses and three subscales of EI (management of others’ emotions, emotional self-awareness, and management of one’s own emotions) and the video test
Fernández-Berrocal et al. (2014)		MSCEIT	PDG	232 psychology students (190 females)	Those with higher EI punctuation had the tendency to score higher on the PDG

*Abbreviations: IGT, Iowa gambling task; IT, inspection time; MSCEIT, Mayer-Salovey-Caruso Emotional Intelligence Test (Mayer et al., 2002); PDG, prisoner’s dilemma game; SJT, Situational Judgement Test (Roberts, 2009); SJTEA, Situational Judgement Test of Emotional Abilities (Roberts et al., 2013); TEMINT, Test of Emotional Intelligence (Schmidt-Atzert and Bühner, 2002); TIE, Emotional Intelligence Test (Test Inteligencji Emocjonalnej; Smieja et al., 2007)*.

Using a decision-making task called the Watson card selection task, Reis et al. (2007) found that those with higher EI (measured by the MSCEIT) exhibited faster RT in the social exchange problems of the cognitive task. Webb et al. (2014) also found a positive correlation between the IGT and MSCEIT total scores and the “facilitating” and “understanding” branches, although they found that EI did not significantly predict the variance of the decision-making task beyond the IQ (cognitive intelligence) scores. In two other studies (which used the simulated arctic rescue scenario and the SJT), Fallon et al. (2013, 2014) did not find any differences between high- and low-EI participants in their cognitive performance, although Fallon et al. (2014) discovered a tendency for EI to correlate with accuracy. Alkozei et al. (2015), using the airport decision-making task mentioned previously, found that higher EI (measured by the MSCEIT) achieved better performance than lower EI in the cognitive task, especially those with higher scores in the “understanding” and “facilitating” branches.

Martin and Thomas (2011) found a negative correlation between RT in a negative-emotional and a neutral-word Stroop task and the MSCEIT, which also accounted for incremental variance beyond a traditional intelligence test. Fiori and Antonakis (2012) did not find any relationship between the MSCEIT and an affective and semantic “priming” task, in which participants had to pay attention to one of two faces (while ignoring the other face), and then categorizing different words that were congruent with either the target or the distractor, or neither of the two.

Dodonova and Dodonov (2010) found a negative correlation between an RT index (the difference between the RT to the emotional face recognition task and the neutral face recognition task) related to the correct responses for the “No” responses in trials where the targets had not yet been presented and three subscales of EI (management of others’ emotions, emotional self-awareness, and management of one’s own emotions) and the video test instrument for EI. Finally, Fernández-Berrocal et al. (2014) found that those with higher EI (measured via the MSCEIT) had a tendency to score higher in the prisoner’s dilemma game (PDG; a decision making task), where participants demonstrate their tendencies to cooperate or compete with other participants.

#### Discussion

**Figure 1:**
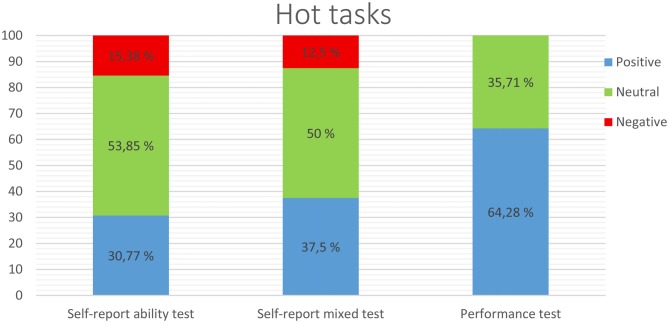
**Relationship between hot cognitive tasks and emotional intelligence (EI) measured through self-report ability tests, self-report mixed tests, or performance tests**.

If we examine the results related to hot cognitive tasks displayed in Figure 1 in a general way, we can see that, using performance tests, EI seems to be more related with positive results. That is, in 64.28% of the studies that used performance tests, participants with better scores in EI obtained better performances in cognitive tasks. When using self-report ability instruments, the results show that higher-EI participants performed better in the cognitive task only in a 30.77% of the studies, followed by 37.5% of positive results with self-report mixed tests. Thus, the percentage of studies finding positive relations between EI and cognitive performance was higher for performance-based ability test than for self-report ability test, *Z* = 15.22, *p* < 0.001, *d* = 0.76, 95% CI [0.68, 0.84], and for self-report mixed text, *Z* = 10.96, *p* < 0.001, *d* = 0.59, 95% CI [0.49, 0.68]. No differences were found between the percentage of positive results between self-report mixed test and self-report ability tests, *Z* = 1.64, *p* = 0.10, *d* = 0.17, 95% CI [0.07, 0.27]. Negative results, in contrast—understood to be worse performances in the cognitive task for higher-EI individuals—were obtained in 15.38% and 12.5% of studies, respectively, via self-report ability tests and mixed tests, and no negative scores were obtained with performance tests. Finally, the studies found no relation between EI and cognitive tasks among 35.71%, 53.85%, and 50% of participants using performance, self-report ability, and mixed tests, respectively.

### Cool Cognitive Tasks

#### Self-report Ability Tests

We identified three studies that used self-report ability tests for measuring EI and cool tasks for the analysis of cognitive processes (Table 4). Austin (2004, 2005) and Farrelly and Austin (2007), in their first experiment, measured the participants’ EI via the EIS, as well as information processing speed via a symbol IT that was similar to the emotional task mentioned previously, but with neutral stimuli. Their results showed no correlation between EI and the symbol IT tasks.

**Table 4:** **Studies using self-report ability EI tests and cool cognitive tasks**.

Study	EI scale	Cognitive task	Sample	Principal results
Austin (2004)	EIS	Symbol IT task	35 department members and	No relationship between EI and IT task
			57 undergraduate students (71 females)	
Austin (2005)	EIS	Symbol IT task	95 adults (71 females)	No relationship between EI and IT task
Farrelly and Austin (2007)	EIS	Symbol IT task	99 university students (70 females)	No relationship between EI and IT task

*Abbreviations: EIS, Schutte Emotional Intelligence Scale (Schutte et al., 1998); IT, inspection time*.

#### Self-report Mixed Tests

We identified three studies that measured EI via self-report mixed instruments (Table 5). Austin (2005) and Farrelly and Austin (2007), in their second experiment, found no relationship between EI assessed via the EQ-i:S and the symbol IT cognitive task. Craig et al. (2009) found a negative correlation between participant performance on a memory task called “digit span” and the BRIEF inventory for EI.

**Table 5:** **Studies using self-report mixed EI tests and cool cognitive tasks**.

Study	EI scale	Cognitive task	Sample	Principal results
Austin (2005)	EQ:i-S	Symbol IT task	95 adults (71 females)	No relationship between EI and IT task
Farrelly and Austin (2007)	EQ:i-S	Symbol IT task	99 university students (70 females)	No relationship between EI and IT task
Craig et al. (2009)	BRIEF	Digit span task	856 participants (446 females)	Negative correlation between performance on the digit span task and the BRIEF

*Abbreviations: BRIEF, Brain Resource Inventory for Emotional Intelligence Factor (Kemp et al., 2005); EQ-i:S, Emotional Quotient Inventory (Bar-On, 2002); IT, inspection time*.

#### Performance Tests

Three experiments were identified for this section (Table 6). In their first study, Farrelly and Austin (2007) found a negative association between a symbol IT task and the “perceiving” branch in the MSCEIT scale, while in their second experiment they did not find any relation between these two. Reis et al. (2007) did not find any correlation between the same EI scale and the descriptive problems of the Watson card task.

**Table 6:** **Studies using performance EI tests and cool cognitive tasks**.

Study		EI scale	Cognitive task	Sample	Principal results
Farrelly and Austin (2007)	Study 1	MSCEIT	Symbol IT task	99 university students (70 females)	Negative association between the symbol IT task and the “perceiving” branch in MSCEIT
Farrelly and Austin (2007)	Study 2	MSCEIT	Discrimination learning task	180 psychology students (111 females)	No relationship between EI and IT task
Reis et al. (2007)		MSCEIT	Watson card selection task: descriptive problems	48 undergraduates students	No relationship between EI and the Watson card selection task

*Abbreviations: IT, inspection time; MSCEIT, Mayer-Salovey-Caruso Emotional Intelligence Test (Mayer et al., 2002)*.

#### Discussion

**Figure 2:**
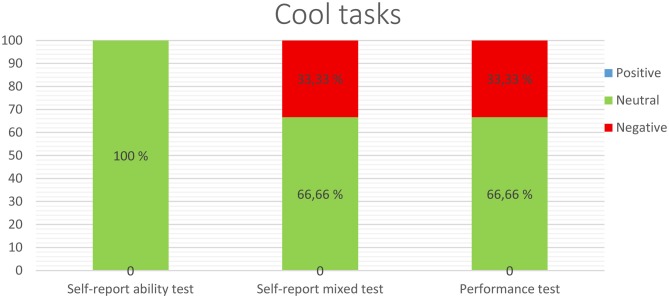
**Relationship between cool cognitive tasks and EI measured through self-report ability tests, self-report mixed tests, or performance tests**.

The overall results from using cool cognitive tasks are displayed in Figure 2; these results demonstrate that no positive relations were found between EI through any of the three perspectives or the tasks. This means that better cognitive performance was not found for higher-EI participants in any of the studies, given that none of the results signal relation between either process. Thus, 100% of the results from the self-report ability tests, and 66.66% of the results from the self-report mixed and performance tests, displayed no relationship with cognitive dimensions. Finally, higher-EI individuals performed worse than lower-EI individuals in those tasks in 33.33% of the self-report mixed and performance tests.

## General Discussion

The present systematic review analyzed the literature on the relationship between EI and cool and hot cognitive processes measured via laboratory tasks rather than traditional tests. We found 26 suitable studies; these used performance EI tests 17 times, self-report ability tests 16 times, and self-report mixed tests 11 times. The 26 studies employed a total of 18 different cognitive tasks, three of which were classified as “cool” and the remaining fifteen as “hot.”

The results of analyzing the hot cognitive tasks showed, as expected, that higher-EI individuals measured via performance tests tended to perform better in these cognitive tasks, while the results were different when using self-report instruments (ability or mixed tests): half of the results point to no relation between either variable. Because “trait EI” is closer to the personality factor, and “performance-based ability EI” to the cognitive process (Webb et al., 2013), it makes sense that we would find relations between EI and cognitive tasks mainly through performance tests, and not with self-report instruments. Specifically, 64% of the results of EI measured with performance tests and cognitive processes were positive, showing an intermediate effect size when compared with self-report measure percentages for positive results.

No positive relations were found for cool cognitive tasks with any EI instrument. The majority of the results showed no relation between EI and cognitive tasks with no emotional content; some results even exhibited worse performance for those with higher EI. These results may reflect the situation that EI favors cognitive performance only when it has emotional information to go on. It is important to note, however, that only nine studies employed cool tasks, in comparison with the 35 instances where hot tasks were used. Thus, future research could be necessary in order to verify this absence of relation.

Although the results related to performance tests and hot cognitive tasks are promising, it is important to note a few limitations of the present study. First, some of the cognitive tasks may have methodological problems from being newly designed instruments whose reliability has not yet been probed; one example is the FDT (Wojciechowski et al., 2014). Second, we observed an enormous variety of instruments that were employed for measuring different aspects of cognition; specifically, 11 different hot cognitive tasks were related with EI performance tests. These measures evaluate distinct cognitive processes such as attention, decision making, and memory, among others; and even if a cognitive task evaluates a specific process, it could be related to different aspects of the process. Thus, the Stroop task (Bar-Haim et al., 2007), for instance, is more centered in the conscious facet of the specific process of attention. All of this variability could affect the results, given that EI could be related to certain cognitive processes but not to others. Finally, a variety of stimuli have been employed; as Bar-Haim et al. (2007) state, the stimulus type is relevant for the results, since faces are more capable of showing bias via threats among anxious people than is the case with fearful words. Apart from these cognitive task–related limitations, five different performance EI tests were employed during the studies we analyzed; these tests are not equally reliable, and they also do not always assess the same dimensions. These factors could have contaminated the results.

In order to make progress in the conceptualization of the EI construct—specifically in understanding the relationship that EI has with hot and cool cognitive processes—it is important to establish future lines of study. Future research should thus be directed toward analyzing the relationship between EI and cognitive-specific tasks (such as attention, memory, and so on). Predictions could vary depending on the kind of cognitive processes that are related with EI. For instance, if we focus on memory processes (and specifically on working memory), we would hope (given the previous literature) that higher-EI individuals would perform better in these cognitive tasks than lower-EI individuals. That prediction, for example, is drawn from the work of Schweizer et al. (2013), who showed via behavioral and brain measures that training on an emotional working memory task improved the emotion regulation ability of the participants; but will higher EI favor performance in, for instance, an attentional cognitive task? Due to the limited number of studies that we would have found by dividing the literature that we reviewed for this study by specific cognitive processes, it will be necessary to address this aspect in future empirical studies in order to provide more insight into how EI interacts with cognition in a wider and more specific manner. It would be also interesting to carry out a future meta-analysis with the data found in order to achieve more precise and robust results. However, given the big variability of dependent variables displayed by the different studies, as well as the few studies of specific cognitive processes found in the revision, a meta-analysis may not be feasible for the current literature.

Another limitation is the absence of studies analyzing the causality of the relations between the variables of interest: All the studies are correlational. It has been shown how EI training can diminish aggressive behavior, negative affect, stress, depression, anxiety, sense of incapacity, as well as promote empathy, wellbeing, health and work performance (Slaski and Cartwright, 2003; Jahangard et al., 2012; Ruiz-Aranda et al., 2012; Castillo et al., 2013). However, can the EI training improve the individual cognitive processing? Get into the answer to this question could favor the implementation of the EI training for covering a wider range of outcomes as the previously mentioned as well as others as the reduction of the age-related cognitive and emotional decline (Cabello et al., 2014) or the attentional, working memory and decision making biases of clinical and non-clinical population (Damasio, 1998; Derakshan and Eysenck, 1998; Mogg and Bradley, 1998; Baddeley, 2007, 2013; Baddeley et al., 2012).

In conclusion, this systematic review contributes to the growing literature on EI and its underlying cognitive processing by suggesting that individuals with higher-EI ability measured through performance tests have advantages in hot cognitive tasks when compared with lower-EI individuals.

## Author Contributions

MJG-C: substantial contributions to the design of the work and interpretation of data for the work; Drafting the work critically for important intellectual conten; Final approval of the version to be published and Agreement to be accountable for all aspects of the work in ensuring that questions related to the accuracy or integrity of any part of the work are appropriately investigated and resolved. RC: substantial contributions to the conception of the work and interpretation of data for the work; Revising the work critically for important intellectual conten; Final approval of the version to be published and Agreement to be accountable for all aspects of the work in ensuring that questions related to the accuracy or integrity of any part of the work are appropriately investigated and resolved. PF-B: substantial contributions to the conception of the work and interpretation of data for the work; Revising the work critically for important intellectual conten; Final approval of the version to be published and Agreement to be accountable for all aspects of the work in ensuring that questions related to the accuracy or integrity of any part of the work are appropriately investigated and resolved.

## Funding

This research was financed by the Spanish Ministry of Economy (PSI2012-37490) and the Innovation and Development Agency of Andalusia, Spain (SEJ-07325).

## Conflict of Interest Statement

The authors declare that the research was conducted in the absence of any commercial or financial relationships that could be construed as a potential conflict of interest.
